# Serum amyloid A in the oral microenvironment: implications for inflammation, host–microbe interactions, and disease monitoring

**DOI:** 10.3389/fimmu.2026.1883479

**Published:** 2026-07-02

**Authors:** Xuefei Cheng, Weichen Gong

**Affiliations:** 1 Department of Microbiology and Immunology, Nihon University School of Dentistry, Matsudo, Japan; 2 Laboratory of Animal Food Function, Graduate School of Agricultural Science, Tohoku University, Sendai, Japan; 3 Livestock Immunology Unit, International Education and Research Center for Food and Agricultural Immunology (CFAI), Graduate School of Agricultural Science, Tohoku University, Sendai, Japan

**Keywords:** inflammation, oral biomarker, oral disease, oral environment, serum amyloid A

## Abstract

**Objective:**

To summarize current evidence regarding the role of serum amyloid A (SAA) in the oral microenvironment, with particular focus on its involvement in oral inflammation, host–microbe interactions, biofilm-associated chronic inflammation, and its potential utility as a diagnostic biomarker.

**Methods:**

Relevant literature regarding SAA in oral inflammation, host–microbe interactions, oral carcinogenesis, and salivary diagnostics was reviewed and synthesized. Particular emphasis was placed on studies investigating oral pathogens, inflammatory signaling pathways, biofilm-associated inflammation, salivary diagnostics, and veterinary applications of salivary SAA monitoring.

**Results:**

Accumulating evidence indicates that SAA is not only a classical acute-phase protein but also an active regulator of inflammatory responses. In the oral cavity, pathogenic microorganisms and persistent biofilm-associated stimulation can induce local and systemic SAA expression through innate immune signaling pathways, including TLR2/TLR4 and NF-κB signaling. SAA contributes to neutrophil recruitment, macrophage activation, cytokine amplification, and maintenance of chronic inflammatory microenvironments. Emerging studies further suggest that SAA may participate in biofilm-associated inflammatory persistence and may contribute to tissue remodeling and disease progression. In addition, SAA can be detected in saliva and inflammatory oral fluids, highlighting its potential as a non-invasive biomarker for disease monitoring. Veterinary studies investigating salivary SAA further support the feasibility of continuous and minimally invasive inflammatory monitoring.

**Conclusions:**

SAA represents an important molecular link between oral microbial dysbiosis, chronic inflammation, and disease progression within the oral microenvironment. Beyond its role as a systemic inflammatory marker, SAA actively participates in immune regulation and host–microbe interactions. Salivary SAA monitoring may provide a promising non-invasive approach for longitudinal assessment of oral inflammatory diseases and related pathological conditions.

## 1 Introduction

The oral cavity represents one of complex microbial habitats in the human body, hosting a diverse community of bacteria, fungi, and viruses that coexist with the host under tightly regulated conditions (1). This dynamic equilibrium is essential for maintaining oral health (2). However, disruption of the oral microbiota, often referred to as dysbiosis, has been implicated in the pathogenesis of various oral diseases, including periodontitis and oral squamous cell carcinoma. Increasing evidence suggests that oral diseases are not solely driven by individual pathogens but rather arise from complex interactions between microbial communities and host immune responses (3).

Serum amyloid A (SAA) is a highly conserved acute-phase protein that is rapidly induced during inflammatory responses. While traditionally regarded as a systemic biomarker of inflammation, accumulating studies have revealed that SAA possesses diverse biological functions, including modulation of innate and adaptive immunity, regulation of cytokine production, and interaction with microbial components (4, 5). Notably, SAA can be induced by bacterial products and has been shown to participate in host defense mechanisms, suggesting a potential role in host–microbe interactions within mucosal environments (5, 6). Although SAA is classically defined as an acute-phase protein, persistent or repeated inflammatory stimuli can lead to sustained elevation of SAA, thereby contributing to chronic inflammatory processes (7).

Chronic inflammation is a central feature linking microbial dysbiosis to disease progression in the oral cavity. Persistent activation of the immune system in response to microbial stimuli can lead to tissue destruction, impaired barrier function, and the establishment of a pro-tumorigenic microenvironment (8). In this context, inflammatory mediators play critical roles not only as markers of disease activity but also as active regulators of disease progression.

In the oral cavity, where microbial exposure is continuous, SAA may act as a critical mediator linking microbial dysbiosis to chronic inflammation (7). Emerging evidence indicates that SAA is not merely a passive indicator but actively contributes to immune cell recruitment, inflammatory signaling amplification, and tissue remodeling. These processes are particularly relevant in the context of oral carcinogenesis, where chronic inflammation and immune dysregulation are recognized as key drivers of tumor initiation and progression (9).

In addition to its mechanistic role, SAA has attracted attention as a potential clinical biomarker (10). The feasibility of detecting SAA in saliva offers a promising non-invasive approach for monitoring oral disease activity and progression. Given the increasing interest in salivary diagnostics, understanding the biological significance of SAA in the oral microenvironment is of considerable importance (11).

In this review, we provide a comprehensive overview of current evidence regarding the role of SAA in oral infection and oral cancer. We focus on its interactions with oral microbiota, its contribution to inflammatory processes, and its potential clinical applications. By integrating these aspects, we aim to highlight SAA as a key mediator within the oral microenvironment and a promising target for future diagnostic and therapeutic strategies.

## 2 Materials and methods

### 2.1 Literature search strategy

A literature search was conducted using PubMed, Web of Science, Scopus, and Google Scholar databases to identify studies related to Serum Amyloid A (SAA) in oral inflammation, host–microbe interactions, oral diseases, and salivary diagnostics. Articles published up to March 2026 were considered.

The search strategy combined terms related to SAA and oral health, including: “Serum Amyloid A”, “SAA”, “oral inflammation”, “periodontitis”, “apical periodontitis”, “oral microbiome”, “oral biofilm”, “oral dysbiosis”, “oral squamous cell carcinoma”, “oral cancer”, “saliva”, and “biomarker”. Boolean operators (AND, OR) were used to optimize the search strategy. Reference lists of relevant articles were also manually screened to identify additional studies not captured through database searches.

### 2.2 Eligibility criteria

Studies were considered eligible if they met one or more of the following criteria: (i) investigated the biological functions or immunological activities of SAA; (ii) examined the relationship between SAA and oral inflammatory diseases, including periodontitis and apical periodontitis; (iii) evaluated host–microbe interactions, microbial dysbiosis, or biofilm-associated inflammation involving SAA; (iv) explored the role of SAA in oral carcinogenesis or oral potentially malignant disorders; or (v) assessed the diagnostic or prognostic utility of salivary SAA.

Original research articles, clinical studies, experimental investigations, and relevant review articles published in English were included. Articles were excluded if they were unrelated to oral diseases or SAA biology, lacked sufficient methodological information, were duplicate records, or were not available in English.

### 2.3 Study selection

All identified records were screened based on titles and abstracts for relevance to the objectives of this review. Potentially eligible articles were subsequently evaluated through full-text assessment. Studies meeting the predefined eligibility criteria were included in the final review.

## 3 Results

The literature search identified a total of 214 records from four databases, including PubMed (n = 100), Web of Science (n = 55), Scopus (n = 35), and Google Scholar (n = 24). After removal of 42 duplicate records, 172 articles remained for title and abstract screening.

During the screening process, 104 records were excluded because they were not directly related to oral diseases, host–microbe interactions, salivary diagnostics, or the biological functions of SAA. The remaining 68 articles underwent full-text evaluation. Following detailed assessment, 10 articles were excluded because they did not meet the predefined eligibility criteria or lacked sufficient relevance to the scope of this review.

Ultimately, 58 studies were included in the qualitative synthesis. These studies were grouped into four thematic categories: (i) biological and immunological functions of SAA, (ii) SAA in oral inflammatory diseases, (iii) host–microbe interactions and biofilm-associated inflammation, and (iv) diagnostic and translational applications of salivary SAA.

**Figure 1:**
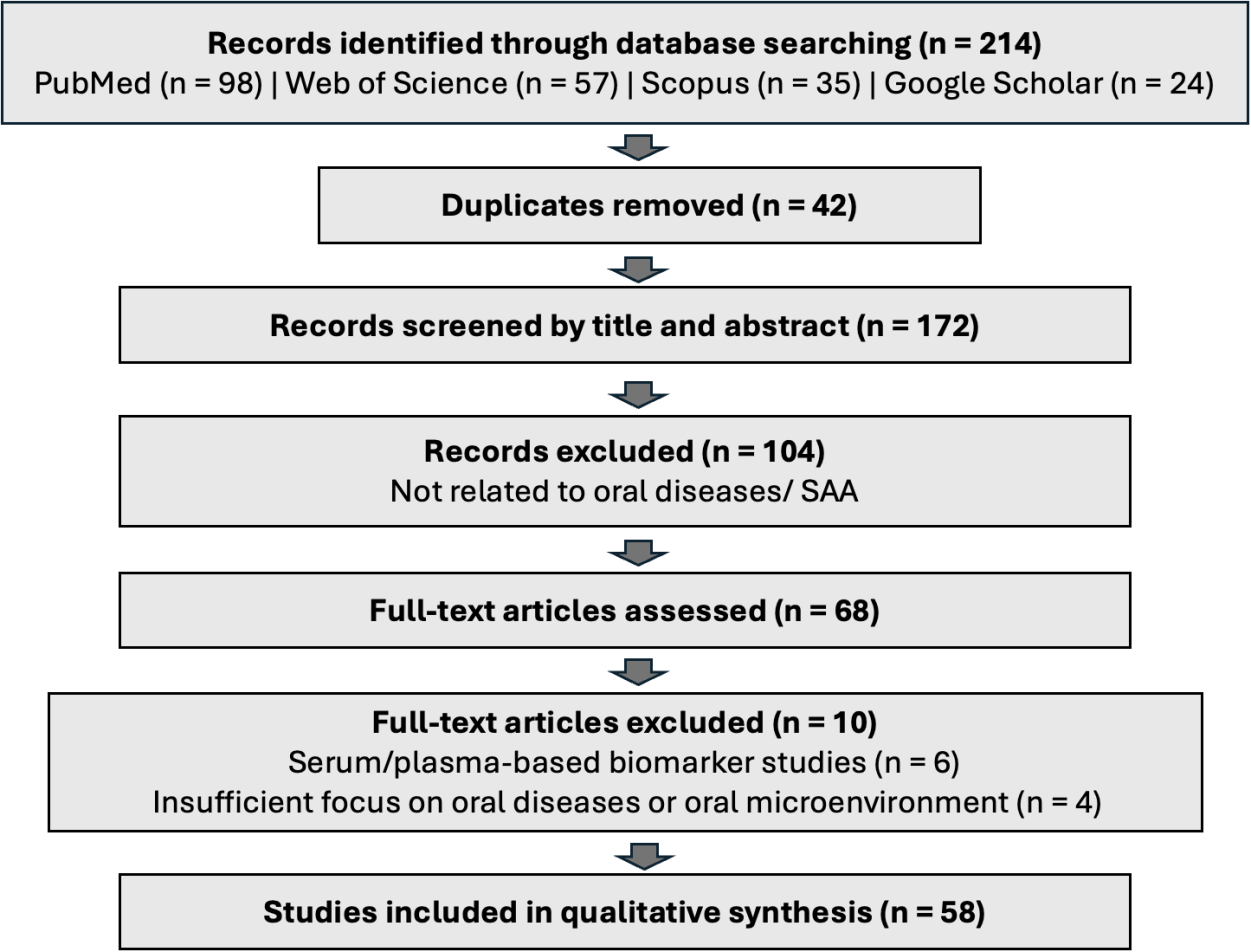
PRISMA flow diagram illustrating the literature search and study selection process. A total of 214 records were identified through database searches. After removal of duplicate records and screening of titles and abstracts, 68 full-text articles were assessed for eligibility. Studies focusing on serum/plasma SAA without specific oral relevance, non-oral disease contexts, or lacking sufficient relevance to the review objectives were excluded. Ultimately, 58 studies were included in the qualitative synthesis.

Additional background references describing general SAA biology, inflammatory signaling pathways, and veterinary applications of salivary SAA were incorporated where necessary to provide mechanistic and translational context. The overall study selection process is summarized in **Figure 1**.

### 3.1 Biology of SAA

SAA is a highly conserved family of acute-phase proteins that plays important roles in inflammation, host defense, and immune regulation. Under physiological conditions, circulating SAA levels remain extremely low; however, during inflammatory responses, infection, tissue injury, or immune activation, SAA expression can increase dramatically, in some cases by more than 1000-fold (12, 13). Traditionally, SAA has been regarded as a classical acute-phase reactant produced mainly by hepatocytes in response to inflammatory cytokines such as interleukin (IL)-1β, IL-6, and tumor necrosis factor (TNF)-α (14). More recent studies, however, have demonstrated that SAA is not merely a passive biomarker of inflammation but also an active regulator of immune responses and tissue homeostasis (15).

In humans, the SAA family consists primarily of four genes, including *SAA1*, *SAA2*, *SAA3*, and *SAA4*. Among them, SAA1 and SAA2 are the major inducible acute-phase isoforms, whereas SAA4 is constitutively expressed. Although human *SAA3* is generally considered a pseudogene, functional SAA3 proteins are expressed in several animal species, particularly in mice, where SAA3 has been implicated in mucosal immunity and epithelial barrier regulation (16). Structurally, SAA proteins are composed of four α-helical domains and a flexible C-terminal region, allowing interaction with a wide range of ligands, including lipids, lipoproteins, bacterial components, and extracellular matrix molecules (17). This structural flexibility contributes to the multifunctional nature of SAA during inflammatory responses.

SAA is strongly associated with innate immune activation. Various microbial products, including lipopolysaccharide (LPS), peptidoglycan, and bacterial outer membrane components, can induce SAA expression through activation of pattern recognition receptors such as Toll-like receptors (TLRs) (4, 18). In particular, TLR2- and TLR4-mediated signaling pathways are important for SAA induction during bacterial infection (19). Downstream activation of nuclear factor kappa B (NF-κB), mitogen-activated protein kinase (MAPK), and signal transducer and activator of transcription 3 (STAT3) pathways further amplifies SAA production (19). In mucosal tissues, epithelial cells themselves may also locally express SAA in response to microbial stimulation, suggesting that SAA participates directly in host–microbe interactions at barrier surfaces (20, 21).

Beyond its induction by bacteria, SAA actively regulates antimicrobial immunity. SAA has been shown to bind outer membrane protein A (OmpA) and related structures on Gram-negative bacteria, thereby promoting bacterial opsonization and enhancing phagocytosis by neutrophils and macrophages (22). In addition, SAA can function as a chemoattractant for immune cells, facilitating the recruitment of neutrophils, monocytes, and T cells to inflammatory sites (23, 24). Through these activities, SAA contributes to the early containment of invading microorganisms and the coordination of innate immune responses.

Importantly, accumulating evidence indicates that SAA also plays major roles in immune modulation during chronic inflammation. Although SAA is classically categorized as an acute-phase protein, persistent microbial stimulation and unresolved inflammation can lead to sustained elevation of SAA expression. Such prolonged induction has been observed in various chronic inflammatory diseases, including inflammatory bowel disease, rheumatoid arthritis, chronic pulmonary inflammation, and cancer-associated inflammation (5, 25–27). In these conditions, SAA functions not only as a marker of inflammation but also as an active amplifier of inflammatory signaling.

One of the most extensively studied mechanisms involves the activation of the NLRP3 inflammasome. SAA has been reported to promote NLRP3 inflammasome activation in macrophages, leading to caspase-1 activation and subsequent secretion of IL-1β and IL-18 (28). This process contributes to amplification of chronic inflammatory responses and maintenance of inflammatory microenvironments. SAA-mediated inflammasome activation is closely linked to NF-κB signaling, which further enhances the transcription of pro-inflammatory cytokines and chemokines (29). Additionally, SAA can induce cyclooxygenase-2 (COX-2) expression and stimulate production of inflammatory mediators such as IL-6, IL-8, and TNF-α, thereby reinforcing inflammatory cascades (29, 30).

SAA also exerts important effects on adaptive immunity. Recent studies have demonstrated that SAA influences T helper cell differentiation, particularly the generation of pathogenic T helper 17 (TH17) cells. SAA can cooperate with IL-6 and other STAT3-activating cytokines to promote differentiation of naïve CD4+ T cells into pro-inflammatory TH17 cells (21). Since TH17-associated immune responses are strongly implicated in chronic inflammatory diseases and tumor-promoting inflammation, sustained SAA signaling may contribute to long-term immune dysregulation (31). Moreover, SAA has been associated with macrophage polarization and regulation of myeloid-derived suppressor cells, further supporting its role in shaping chronic inflammatory microenvironments (23).

In addition to its pro-inflammatory functions, SAA also exhibits protective and tissue repair-associated properties (7). SAA can bind high-density lipoprotein (HDL) and participate in lipid transport during inflammation, facilitating clearance of membrane debris and toxic lipids generated during tissue damage (32). SAA has also been implicated in epithelial barrier maintenance and antimicrobial peptide induction in mucosal tissues (33). These findings suggest that SAA possesses a dual role during inflammation, functioning both as a mediator of host defense and as a contributor to chronic inflammatory pathology depending on the inflammatory context and duration of stimulation.

Persistent elevation of SAA has further been associated with pathological amyloid deposition and chronic inflammatory complications. Long-term overproduction of SAA is a major driver of AA amyloidosis, highlighting the pathological consequences of sustained SAA activation (17, 34). Emerging evidence also suggests that SAA may contribute to chronic inflammation-associated tumorigenesis through modulation of cytokine signaling, immune cell recruitment, angiogenesis, and tissue remodeling (27). Collectively, these findings indicate that SAA serves as a multifunctional inflammatory mediator linking microbial stimulation, immune activation, chronic inflammation, and tissue pathology.

### 3.2 SAA in oral diseases

**Figure 2:**
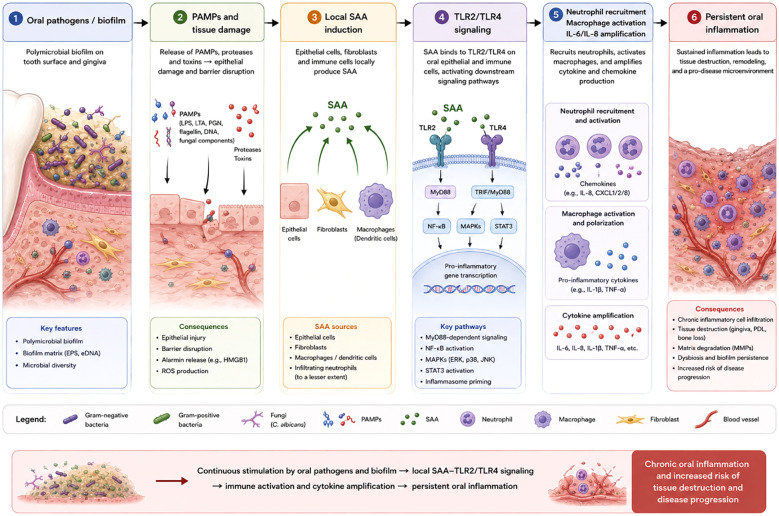
Proposed model of SAA-mediated chronic inflammation in the oral microenvironment. Persistent oral pathogens within polymicrobial biofilms continuously stimulate epithelial and immune cells through pathogen-associated molecular patterns (PAMPs), microbial toxins, and tissue damage signals. These stimuli induce local production of serum amyloid A (SAA) by gingival epithelial cells, fibroblasts, macrophages, and other inflammatory cells within the oral microenvironment. SAA subsequently activates TLR2/TLR4-dependent signaling pathways, including MyD88, NF-κB, MAPKs, and STAT3 signaling, resulting in amplification of pro-inflammatory gene expression. Downstream effects include neutrophil recruitment and activation, macrophage activation and polarization, and increased production of inflammatory cytokines and chemokines such as IL-6, IL-8, IL-1β, TNF-α, and CXCL family members. Persistent activation of these inflammatory pathways contributes to chronic oral inflammation, tissue destruction, extracellular matrix degradation, and maintenance of dysbiotic biofilms, thereby creating a pro-disease microenvironment associated with progression of chronic oral inflammatory diseases. This schematic illustration was created with AI-assisted image generation and subsequently refined and scientifically validated by the authors.

Oral infections are typically driven by polymicrobial biofilms rather than by single pathogens. Persistent colonization by oral bacteria and fungi provides repeated microbial stimulation to epithelial, stromal, and immune cells, thereby maintaining a chronic inflammatory microenvironment (35). In this context, SAA may act as both a marker and mediator of oral inflammation. Although SAA was originally characterized as a systemic acute-phase protein, evidence from periodontal disease and apical periodontitis indicates that SAA can also be locally elevated in oral inflammatory lesions and may contribute to the persistence of inflammation (36) (**Figure 2**).

#### 3.2.1 Evidence of SAA expression in oral inflammatory diseases

Clinical studies have reported increased SAA levels in oral inflammatory conditions. In patients with gingivitis and chronic periodontitis, SAA levels in both gingival crevicular fluid and serum were significantly higher than in healthy controls, and gingival crevicular fluid SAA correlated with periodontal pocket depth in chronic periodontitis (37, 38). This suggests that SAA may reflect both local periodontal inflammation and systemic inflammatory burden.

More direct mechanistic evidence comes from chronic apical periodontitis. Hirai et al. analyzed human periapical surgical specimens and experimental mouse models and showed that SAA1/2 was locally expressed in human periapical lesions at both mRNA and protein levels (11). SAA protein levels appeared to correlate with the inflammatory status of lesions. In mouse periapical inflammation, SAA1.1/2.1 and SAA3 were elevated locally and systemically. SAA-deficient mice showed reduced inflammatory cell infiltration, and recombinant human SAA1 directly induced neutrophil chemotaxis, prolonged neutrophil survival, and activated macrophage IL-1α production through TLR2/TLR4-dependent signaling (11).

These findings are important because they support a model in which SAA is not merely a circulating biomarker leaking into inflamed oral tissues, but may be produced or maintained locally within oral inflammatory lesions. The oral cell types currently implicated include macrophages in periapical lesions, human gingival fibroblasts, and gingival epithelial compartments. In inflamed gingiva, SAA expression is increased, and SAA can stimulate human gingival fibroblasts to produce inflammatory cytokines through the TLR2 pathway.

#### 3.2.2 Relationship between major oral pathogens and SAA

Among oral pathogens, *Porphyromonas gingivalis* currently has the strongest experimental evidence linking oral infection to SAA-associated inflammatory responses. In contrast, evidence involving *Fusobacterium nucleatum* and *Streptococcus mutans* remains largely indirect and is primarily based on their established roles in chronic inflammation, biofilm persistence, and oral carcinogenesis (**Table 1**).

**Table 1:** Oral bacteria and fungi associated with oral cancer.

Microorganism	Type	Reported cancer association	Main evidence	Possible mechanisms	Reference(s)
*Porphyromonas gingivalis*	Gram-negative anaerobic bacterium	OSCC; oro-digestive cancers	Human association studies; *in vitro* oral cancer cell studies; mouse tumor/inflammation models	TLR activation, chronic inflammation, gingipains, immune evasion, epithelial invasion, cancer stemness, enhanced proliferation/invasion	(39, 40)
*Fusobacterium nucleatum*	Gram-negative anaerobic bacterium	OSCC; also strongly linked to colorectal cancer	Human microbiome studies; tumor-associated enrichment; mechanistic studies on FadA	Adhesion via FadA, β-catenin/NF-κB signaling, EMT, immune modulation, biofilm bridging, amyloid-like FadA	(41–43)
*Streptococcus mutans*	Gram-positive facultative bacterium	OSCC	Human tumor colonization data; oral cancer cell and xenograft models; 4NQO mouse model	IL-6 induction, EMT, MDSC recruitment, metabolic reprogramming of tumor microenvironment	(44–46)
*Candida albicans*	Fungus	OSCC; oral potentially malignant disorders	Epidemiological association; *in vitro* and animal studies	Acetaldehyde production, epithelial invasion, biofilm formation, chronic inflammation, immune evasion	(56, 57)

For *P. gingivalis*, animal data provide the strongest link to systemic SAA responses. In a chronic oral infection model using ApoE-null mice, oral infection with *P. gingivalis* induced alveolar bone loss and viable bacterial invasion of oral epithelium and aortic tissue. After 24 weeks of infection, mice showed significantly elevated serum amyloid A compared with controls, indicating that chronic oral *P. gingivalis* infection can drive systemic inflammatory responses involving SAA (39).

In addition, *P. gingivalis* outer membrane vesicles have recently been reported to suppress osteogenic differentiation of bone marrow mesenchymal stem cells through an SAA3-mediated TLR4/MyD88/NF-κB axis, suggesting that *P. gingivalis*-derived components may interact with SAA-related inflammatory signaling (40). However, this evidence is based mainly on experimental systems and should be distinguished from direct human oral lesion data. Although these findings support a relationship between *P. gingivalis* infection and SAA-associated inflammatory responses, direct evidence demonstrating local SAA induction within human periodontal lesions remains limited.

For *F. nucleatum*, direct evidence connecting this bacterium to SAA in the oral cavity remains limited. Nevertheless, *F. nucleatum* is strongly associated with oral biofilm maturation, periodontal inflammation, and oral squamous cell carcinoma (41). Its virulence factors, including FadA, can promote epithelial adhesion, inflammatory activation, and tumor-associated signaling (42). Importantly, *F. nucleatum* can form amyloid-like FadA structures that contribute to pathogenicity, periodontal bone loss, and cancer-related phenotypes (43). This makes *F. nucleatum* highly relevant to a broader SAA–biofilm–amyloid framework, even if direct induction of SAA by *F. nucleatum* in oral tissues has not yet been fully established. Therefore, the relationship between *F. nucleatum* and SAA should currently be regarded as biologically plausible but not experimentally established.

For *S. mutans*, the direct SAA literature is also limited. However, *S. mutans* is a key cariogenic bacterium and an important biofilm-forming species (44). Recent studies suggest that *S. mutans* may also participate in oral squamous cell carcinoma progression (45). One study reported that *S. mutans* infection enhanced tumor aggressiveness, epithelial–mesenchymal transition, IL-6 production, and myeloid-derived suppressor cell recruitment in oral cancer models (45). Another study further showed that tumor-colonized *S. mutans* can metabolically reprogram the tumor microenvironment and promote oral squamous cell carcinoma (OSCC) progression (46). At present, no direct studies have demonstrated that *S. mutans* induces SAA expression in oral tissues.

Taken together, current evidence supports a cautious but biologically plausible model: major oral pathogens may not all have been directly shown to induce SAA, but they can generate persistent inflammatory and biofilm-associated stimulation. This persistent stimulation may induce local or systemic SAA responses, which in turn amplify immune activation and contribute to chronic oral inflammation.

#### 3.2.3 SAA-mediated immune responses in oral infection

SAA can influence oral infection through several immune mechanisms. First, SAA promotes neutrophil recruitment. In experimental periapical inflammation, recombinant SAA1 induced neutrophil chemotaxis in a dose-dependent manner, and TLR2/TLR4 deficiency reduced this response (47, 48). SAA also prolonged neutrophil survival, which may contribute to sustained inflammatory cell infiltration in chronic lesions (49).

Second, SAA activates macrophages. In the same experimental system, SAA stimulated macrophage IL-1α production through TLR2/TLR4-dependent signaling (49). This finding is highly relevant to oral inflammatory lesions because macrophages are abundant in chronic periodontitis, apical periodontitis, and tumor-associated inflammatory microenvironments.

Third, SAA amplifies cytokine production. In human gingival fibroblasts, SAA increased IL-6 and IL-8 expression, and this effect was suppressed by blocking TLR2 (50). Since IL-6 and IL-8 are closely associated with periodontal inflammation, neutrophil recruitment, angiogenesis, and tumor-promoting inflammation (51), the SAA–TLR2–IL-6/IL-8 axis may represent an important mechanism linking oral infection to chronic inflammatory remodeling.

#### 3.2.4 SAA, oral biofilms, and chronic inflammatory persistence

Oral infections are frequently biofilm-associated. Dental plaque is a polymicrobial biofilm embedded in a matrix of bacterial polymers and host-derived salivary components (52, 53). The acquired pellicle and plaque matrix contain proteins from saliva, gingival crevicular fluid, blood, mucosa, bacteria, and diet, creating a complex interface between host proteins and microbial communities.

SAA may enter this interface from serum, gingival crevicular fluid, saliva, or local inflammatory tissues. Although direct mechanistic studies showing that SAA enhances oral biofilm formation remain insufficient, its presence in inflammatory oral fluids and its ability to interact with bacterial components make this a reasonable hypothesis (54). In a biofilm-rich environment, SAA could potentially influence microbial adhesion, immune recognition, or matrix composition. Conversely, persistent biofilms may repeatedly induce SAA expression through bacterial PAMPs and tissue damage signals. From a mechanistic perspective, several potential pathways may connect SAA with oral biofilm biology. SAA has been shown to bind bacterial outer membrane proteins such as OmpA and can interact with extracellular matrix-associated molecules (22). These observations raise the possibility that SAA may influence bacterial adhesion, host-protein incorporation into biofilms, or immune recognition at biofilm interfaces. Furthermore, because oral biofilms contain substantial amounts of host-derived proteins, extracellular DNA (eDNA), and inflammatory exudates, SAA may become incorporated into biofilm-associated matrices under inflammatory conditions (54). However, direct evidence demonstrating effects of SAA on oral biofilm architecture, extracellular polymeric substance composition, microbial adhesion, or biofilm maturation is currently unavailable.

This concept is particularly relevant to oral carcinogenesis. Long-term colonization by pathogenic biofilms may generate repeated cycles of microbial stimulation, epithelial damage, neutrophil infiltration, macrophage activation, and cytokine production (55). SAA may amplify this process by sustaining TLR-dependent inflammatory responses. Over time, this chronic inflammatory niche may promote epithelial barrier disruption, tissue remodeling, oxidative stress, and tumor-promoting immune responses.

#### 3.2.5 Oral fungal infection and SAA

Compared with bacterial oral infections, the relationship between oral fungal infection and SAA is less well characterized. *Candida albicans* is the most common cause of oral candidiasis and can form biofilms, invade epithelial tissues, and interact with oral bacteria (56). Although oral-specific studies on salivary or local SAA in candidiasis remain limited, experimental work outside the oral cavity suggests that SAA can be induced during *C. albicans* infection and may exert antifungal activity. One study demonstrated that mammalian SAA1 increased after *C. albicans* infection and showed antifungal activity against *C. albicans* (57).

Therefore, in the oral cavity, SAA may potentially participate in antifungal host defense, but this remains an underexplored area. Given the known association between chronic candidiasis, oral potentially malignant disorders, and OSCC, future studies should examine whether SAA contributes to host defense, persistent inflammation, or epithelial remodeling during oral Candida infection.

### 3.3 Diagnostic and translational applications of SAA

The development of reliable, non-invasive biomarkers remains an important challenge in oral medicine. Conventional blood-based inflammatory markers require repeated venipuncture and are therefore not ideal for continuous monitoring of chronic oral diseases. In contrast, saliva represents an attractive diagnostic fluid because it can be collected non-invasively, repeatedly, and with minimal discomfort. Saliva also directly reflects the local oral microenvironment, including microbial composition, mucosal inflammation, gingival crevicular fluid components, and tissue-derived inflammatory mediators. In this context, SAA has emerged as a promising candidate biomarker for inflammatory disease monitoring (**Table 2**).

**Table 2:** Reported clinical and veterinary applications of SAA detection.

Application field	Sample type	Disease/condition	Main findings	Clinical significance	Reference(s)
Chronic periodontitis	Gingival crevicular fluid/serum	Chronic periodontitis	Increased SAA correlated with periodontal severity	Biomarker for periodontal inflammation	(54)
Apical periodontitis	Periapical tissue/serum	Chronic apical periodontitis	Local SAA expression associated with inflammatory lesions	Indicator of active oral inflammation	(11)
Oral squamous cell carcinoma (OSCC)	Saliva	OSCC	Acute-phase proteins altered in saliva proteomics	Potential non-invasive biomarker panel	(45, 46)
Companion animals (dogs/cats)	Serum	Infection, neoplasia, inflammatory disease	SAA rises rapidly during inflammation	Monitoring inflammation and treatment response	(62)
Horses	Serum/saliva	Systemic inflammation, stress	SAA highly sensitive to inflammatory activity	Early detection and disease monitoring	(63)
Cattle	Serum/saliva	Mastitis and inflammatory disease	Elevated SAA associated with infection severity	Herd health management	(64)
Pigs	Saliva	Stress and inflammatory responses	Salivary SAA measurable and responsive to disease/stress	Non-invasive monitoring in livestock	(64, 65)

Traditionally, SAA has been measured in serum or plasma as a highly sensitive acute-phase protein (58). Elevated circulating SAA levels have been reported in systemic inflammatory diseases, bacterial infections, autoimmune diseases, and cancer (59). More recently, SAA has also been detected in saliva, gingival crevicular fluid, and oral inflammatory lesions, raising interest in its application as a non-invasive biomarker for oral diseases (60). Importantly, unlike transient inflammatory cytokines such as IL-6, SAA often shows more stable and sustained elevation during ongoing inflammatory stimulation, potentially making it suitable for longitudinal disease monitoring.

#### 3.3.1 Clinical studies of SAA in oral diseases

Several clinical studies have reported elevated SAA levels in oral inflammatory conditions. In chronic periodontitis, gingival crevicular fluid SAA concentrations were significantly increased and positively correlated with probing pocket depth and clinical attachment loss (38). Serum SAA levels were also elevated in patients with severe periodontal inflammation, suggesting that local oral infection can contribute to systemic inflammatory burden.

Similarly, studies on apical periodontitis demonstrated increased local expression of SAA in inflamed periapical tissues, supporting the possibility that SAA reflects active inflammatory lesions in the oral cavity (11). Experimental evidence further suggests that SAA participates directly in lesion progression through neutrophil recruitment and macrophage activation rather than acting solely as a passive biomarker (61).

In oral cancer, interest in inflammatory biomarkers has expanded rapidly due to the close relationship between chronic inflammation and carcinogenesis. Although direct clinical studies specifically evaluating salivary SAA in OSCC remain limited, proteomic analyses of saliva from OSCC patients have repeatedly identified acute-phase proteins and inflammatory mediators as altered components. These findings suggest that SAA may potentially serve as part of a multi-marker inflammatory panel for oral cancer detection and disease monitoring.

#### 3.3.2 Salivary SAA as a non-invasive biomarker

One of the major advantages of salivary SAA detection is the feasibility of repeated sampling. Chronic oral diseases such as periodontitis and oral potentially malignant disorders often progress slowly and fluctuate over time. Therefore, continuous monitoring rather than single time-point testing may provide more clinically meaningful information regarding disease activity and treatment response.

Compared with blood collection, saliva sampling offers several advantages: (a) non-invasive collection; (b) minimal patient discomfort; (c) suitability for repeated or home-based monitoring; (d) lower cost and easier large-scale screening; (e) direct reflection of oral inflammatory conditions.

These characteristics make salivary SAA particularly attractive for future point-of-care testing (POCT) systems and wearable biosensor-based diagnostics. Emerging microfluidic and biosensor technologies may further improve the sensitivity and reproducibility of salivary SAA detection.

Importantly, salivary biomarkers may reflect both local and systemic inflammation. Because oral inflammatory diseases are closely linked to microbial dysbiosis and chronic mucosal immune activation, salivary SAA could potentially function as an integrated readout of microbial stimulation and host inflammatory responses.

Despite these advantages, several factors may influence salivary SAA measurements. Circadian variation, recent food intake, smoking status, oral hygiene practices, hydration status, medication use, and concurrent systemic inflammation may all affect biomarker concentrations. In addition, differences in saliva collection methods, processing procedures, storage conditions, and analytical platforms may contribute to inter-study variability.

#### 3.3.3 Veterinary evidence supporting salivary SAA monitoring

Veterinary medicine has provided important evidence supporting the clinical utility of salivary SAA monitoring. In companion animals and livestock, SAA has already been widely applied as a practical inflammatory biomarker.

In dogs and cats, serum SAA is routinely used for monitoring infection, inflammatory disease, and postoperative recovery. Elevated SAA levels have been reported in bacterial infections, neoplastic disease, and systemic inflammatory conditions (62). In horses, SAA is considered one of the most sensitive acute-phase proteins for detecting inflammatory disorders and monitoring treatment response (63). In cattle and pigs, SAA measurement has been explored for mastitis, respiratory disease, and herd health management (64).

Importantly, salivary SAA measurement has also attracted increasing attention in veterinary diagnostics. Salivary SAA have been investigated as stress and inflammation biomarkers in pigs because saliva collection is significantly less stressful than blood sampling (65). These veterinary studies support the broader feasibility of saliva-based SAA monitoring and highlight its potential translational value for human oral medicine.

#### 3.3.4 Future perspectives for continuous SAA monitoring

Current evidence suggests that SAA may have utility not only as a diagnostic biomarker but also as a dynamic indicator of disease progression. Since oral diseases are characterized by repeated microbial stimulation and fluctuating inflammatory activity, continuous monitoring strategies may provide greater clinical value than single measurements.

Future developments may include: (a) longitudinal salivary SAA monitoring for periodontitis progression; (b) integration of SAA with oral microbiome profiling; (c) multiplex biomarker panels combining SAA with IL-6, CRP, or microbial markers; (d) biosensor-based real-time oral inflammatory monitoring; (e) saliva-based screening systems for oral potentially malignant disorders and OSCC.

Because SAA can reflect both inflammatory activation and host–microbe interactions, combining salivary SAA detection with oral microbiota analysis may provide a more comprehensive assessment of disease status. Such an approach could help identify high-risk inflammatory microenvironments before irreversible tissue destruction or malignant transformation occurs.

Nevertheless, several limitations remain. Salivary SAA concentrations can be influenced by systemic inflammation, oral hygiene status, smoking, diet, and sampling conditions. In addition, standardized collection methods and diagnostic cut-off values are not yet fully established. Therefore, large-scale longitudinal studies are still required before routine clinical implementation can be achieved.

Another unresolved issue is the comparative diagnostic performance of SAA relative to established oral inflammatory biomarkers. Biomarkers such as IL-6, IL-8, CRP, matrix metalloproteinases (MMP-8 and MMP-9), and gingival crevicular fluid-derived inflammatory mediators have been more extensively investigated in oral disease settings (37, 38). Direct comparative studies evaluating sensitivity, specificity, predictive value, and longitudinal responsiveness are still lacking for salivary SAA.

Collectively, current evidence suggests that SAA represents a promising biomarker linking oral inflammation, microbial dysbiosis, and disease progression. The feasibility of salivary SAA detection further highlights its potential as a non-invasive and continuously monitorable indicator for future oral disease diagnostics.

## 4 Discussion

Accumulating evidence indicates that SAA is more than a classical acute-phase protein and should instead be regarded as a multifunctional mediator linking microbial stimulation, immune activation, chronic inflammation, and tissue pathology (9). In the oral cavity, where microbial exposure is continuous and biofilm formation is persistent, SAA may play particularly important roles in shaping inflammatory microenvironments. Current studies suggest that oral pathogens and chronic oral infections can induce local and systemic SAA responses, while SAA itself contributes to neutrophil recruitment, macrophage activation, cytokine amplification, and sustained inflammatory signaling through pathways including TLR2/TLR4, NF-κB, STAT3, and NLRP3 inflammasome activation (9).

Importantly, oral diseases are increasingly recognized as polymicrobial and biofilm-associated disorders rather than infections caused by single pathogens alone. This concept is highly relevant to SAA biology. Persistent biofilms composed of bacteria, fungi, eDNA, host proteins, and amyloid-like structures may repeatedly stimulate epithelial and immune cells, thereby maintaining prolonged SAA induction (54). In turn, sustained SAA signaling may reinforce inflammatory feedback loops and contribute to chronic tissue remodeling. Such chronic inflammatory niches are now considered important drivers of oral carcinogenesis. While chronic inflammation is widely recognized as a driver of oral carcinogenesis, direct evidence demonstrating a causal role for SAA in OSCC development remains limited. Current evidence is largely derived from indirect associations and studies performed in non-oral cancer models.

However, SAA does not function exclusively as a pathogenic mediator. Several studies have demonstrated protective roles for SAA in mucosal immunity, epithelial barrier maintenance, antimicrobial defense, and tissue repair. Furthermore, correlations between SAA levels and disease severity have not been uniformly observed across all clinical studies. These findings suggest that SAA functions may be context-dependent and influenced by tissue type, disease stage, microbial composition, and host immune status. Therefore, SAA should not be viewed solely as a driver of pathology but rather as a multifunctional immune mediator with both protective and potentially pathogenic properties.

Although direct mechanistic studies remain limited, emerging findings support a potential relationship between SAA and oral biofilm-associated pathology. SAA has already been detected in inflammatory oral fluids and lesions, and several oral pathogens associated with biofilm persistence and oral cancer, including *P. gingivalis*, *F. nucleatum*, *S. mutans*, and *C. albicans*, are capable of promoting chronic inflammatory signaling and epithelial dysregulation (66). Future studies should therefore investigate whether SAA directly interacts with oral biofilm components, including bacterial amyloid proteins and extracellular matrix structures, and whether these interactions contribute to persistent inflammation and tumor-promoting microenvironments.

Another important perspective is the application of SAA as a diagnostic biomarker. Compared with conventional blood sampling, saliva-based biomarker detection provides several practical advantages, including non-invasive collection, repeatability, reduced patient burden, and suitability for longitudinal monitoring. Evidence from both human and veterinary medicine supports the feasibility of salivary SAA detection as a marker of inflammatory activity. In chronic oral diseases, where disease activity often fluctuates over long periods, continuous monitoring of salivary SAA may provide clinically valuable information regarding disease progression, treatment response, and recurrence risk. Despite these promising findings, several important limitations should be considered when interpreting the current literature and assessing the potential clinical utility of salivary SAA. First, the number of well-controlled human clinical studies directly evaluating salivary SAA in oral diseases remains limited. Most available evidence originates from experimental models or studies of periodontal disease, whereas data from oral potentially malignant disorders and oral squamous cell carcinoma are scarce. Second, SAA is a non-specific inflammatory biomarker that may be influenced by systemic inflammatory conditions unrelated to oral disease. Third, standardized saliva collection protocols, reference ranges, and clinically validated diagnostic cut-off values for salivary SAA have not yet been established. These limitations currently restrict the routine clinical implementation of salivary SAA and highlight the need for large-scale longitudinal validation studies.

Future studies should also focus on integrating SAA detection with oral microbiome analysis. Since oral diseases arise through complex interactions between microbial communities and host immunity, combining microbial profiling with inflammatory biomarkers may provide more accurate and dynamic disease assessment than either approach alone. Advances in biosensor technology, microfluidics, and point-of-care testing systems may further facilitate the development of real-time oral inflammatory monitoring platforms based on salivary SAA.

To advance the field, future research should move beyond simply documenting elevated SAA levels in oral diseases and focus on defining the functional role of SAA within the oral microenvironment. A central unresolved question is whether SAA acts primarily as a biomarker reflecting ongoing inflammation or as an active effector that shapes host–microbe interactions and disease progression.

At present, evidence demonstrating direct effects of SAA on oral microbial communities, biofilm architecture, or microbial virulence remains scarce. Likewise, it is unclear whether SAA contributes to tissue pathology through amplification of chronic inflammation or whether it primarily functions as a protective mediator that supports antimicrobial defense and tissue homeostasis. Resolving these questions will require mechanistic studies combining microbiome analysis, biofilm models, and host immune profiling.

Another major challenge is determining whether salivary SAA provides clinically meaningful information beyond existing inflammatory biomarkers. Rather than focusing solely on diagnostic performance, future studies should investigate whether longitudinal SAA monitoring can predict disease progression, treatment response, or malignant transformation before overt clinical deterioration becomes apparent.

Ultimately, a better understanding of how SAA integrates microbial signals with host immune responses may establish SAA not merely as a marker of oral inflammation but as a central regulator of oral immune homeostasis.

## 5 Conclusions

In conclusion, SAA represents a promising molecular link connecting oral microbial dysbiosis, host immune responses, chronic inflammation, and oral disease progression. Emerging evidence suggests that SAA is not merely a systemic acute-phase biomarker but also an active participant in host–microbe interactions within the oral microenvironment. Through its effects on neutrophil recruitment, macrophage activation, cytokine amplification, and inflammatory signaling pathways, SAA may contribute to the maintenance of chronic inflammatory conditions associated with periodontal disease, apical periodontitis, and potentially oral carcinogenesis.

Although direct mechanistic evidence remains limited in several areas, current findings support a model in which persistent microbial stimulation and biofilm-associated inflammation promote sustained SAA expression, which may in turn influence local immune responses and tissue remodeling. At the same time, accumulating data indicate that SAA may also exert protective functions related to antimicrobial defense and maintenance of mucosal homeostasis, highlighting its context-dependent and multifunctional nature.

From a translational perspective, the detection of SAA in saliva offers an attractive opportunity for non-invasive disease monitoring. Future integration of salivary SAA measurement with microbiome profiling, multiplex biomarker panels, and emerging biosensor technologies may improve the diagnosis and longitudinal assessment of oral inflammatory diseases and oral potentially malignant conditions.

Ultimately, further mechanistic, clinical, and longitudinal studies will be required to determine whether SAA functions primarily as a biomarker, an effector molecule, or both. A deeper understanding of SAA biology may not only advance our knowledge of oral inflammation and carcinogenesis but also facilitate the development of novel diagnostic and therapeutic strategies in oral medicine.
